# Effect of Al_2_O_3_ and SiC Fillers on the Structure and Properties of UHMWPE-Based Composite Coatings Produced by Flame Spraying

**DOI:** 10.3390/polym18151879

**Published:** 2026-07-30

**Authors:** Mazhyn Skakov, Perassyl Zhanimkhan, Danel Skakov, Dastan Buitkenov, Meruyert Maulet, Aiym Nabioldina

**Affiliations:** 1 Department of Physics and Technologies, Sarsen Amanzholov East Kazakhstan University, Ust-Kamenogorsk 070000, Kazakhstan; skakovnnc@gmail.com (M.S.); dbuitkenov@vku.edu.kz (D.B.); maulet_meruert@mail.ru (M.M.); anabioldina@vku.edu.kz (A.N.); 2 Agency of the Republic of Kazakhstan for Atomic Energy, National Nuclear Center of the Republic of Kazakhstan, Kurchatov 071100, Kazakhstan; 3 Altay Polymetals LLP, Almaty 100835, Kazakhstan; d.skakov@altaypm.kz

**Keywords:** UHMWPE, Al_2_O_3_ coating, SiC coating, composite coating, flame spraying, microhardness, abrasive wear

## Abstract

This study investigated the structure and properties of flame-sprayed composite coatings based on ultra-high molecular weight polyethylene (UHMWPE) modified with Al_2_O_3_ and SiC ceramic fillers. The results of the composite coatings showed that the filler particles were preserved within the polymer matrix, covered by polymer fibers, and strongly bonded to the matrix. The X-ray diffraction analysis showed that for the samples containing Al_2_O_3_, the degree of crystallinity decreased from 77% to 64%, while for the SiC-containing samples, it remained in the range of 71–76%. The maximum microhardness was achieved in the coating with 15 wt.% Al_2_O_3_, reaching 8.15 ± 0.5 HV0.03, which represents a 50.9% increase compared with the initial UHMWPE coating. In the case of the SiC filler, the maximum microhardness was achieved at a content of 20 wt.%, reaching 7.40 ± 0.4 HV0.03. The abrasive wear test results demonstrated that both fillers enhanced the wear resistance of the coatings. The coatings containing 15–20 wt.% SiC exhibited the highest wear resistance, with a mass loss of approximately 0.02 g. In the case of the Al_2_O_3_ filler, the minimum mass loss was achieved at a content of 20 wt.%, reaching approximately 0.032 g.

## 1. Introduction

At present, increasingly stringent requirements are being imposed on the reliability and durability of technological equipment used in the mining, non-ferrous metallurgy, and petrochemical industries. Pipelines, pumps, storage tanks, conveyor systems, and various technological units used in these industries are exposed to aggressive environments, mechanical loads, abrasive particles, and corrosive factors during operation. In particular, in non-ferrous metallurgy enterprises, equipment is exposed to intensive abrasive wear during ore crushing, beneficiation, and hydraulic transportation processes, while in the oil and gas and petrochemical industries, the influence of corrosive media remains one of the key challenges. Therefore, the development of highly efficient protective coatings for metal structures operating under these conditions represents an important scientific and engineering task. At present, polymer coatings based on epoxy, polyurethane, polyethylene, and polyaniline are widely used for the protection of metal surfaces [1,2,3]. These materials are distinguished by their relatively low cost, chemical stability, and corrosion protection capability. However, the increasing severity of modern industrial operating conditions, including high mechanical loads, friction processes involving hard particles, and aggressive environments, results in the premature degradation of conventional polymer coatings [4]. In this context, the development of next-generation composite coatings with high mechanical strength, wear resistance, and long-term durability in corrosive environments has become necessary.

Ultra-high molecular weight polyethylene (UHMWPE) is considered one of the promising materials. This polymer exhibits high impact resistance, a low friction coefficient, and excellent wear resistance. Furthermore, UHMWPE is widely used in the medical field owing to its biological inertness. Nevertheless, the high viscosity and relatively low load-bearing capacity of UHMWPE restrict its engineering applications to a certain extent. In recent years, the modification of UHMWPE with various micro- and nanofillers has been actively investigated as an approach to improving its properties. In particular, the incorporation of carbon nanotubes (CNTs), graphene and graphene oxide, nanoclays, aluminum oxide (Al_2_O_3_), silicon carbide (SiC), and other inorganic particles is regarded as an effective strategy for enhancing the mechanical, tribological, and performance properties of the material. According to literature reports, the addition of 0.2 wt.% single-walled carbon nanotubes can enhance the mechanical properties of UHMWPE composite coatings by approximately 66% [5]. The studies conducted by S.R. Bakshi et al. [6] demonstrated that the incorporation of 4 wt.% carbon nanotubes into UHMWPE coatings ensured uniform filler dispersion, increased the crack resistance of the coating by 56%, and enhanced the degree of crystallinity from 53.7% to 80.4%. The study by A.S. Mohammed [7] showed that the UHMWPE composite coating with 3 wt.% Al_2_O_3_ demonstrated the best mechanical and tribological performance. Jing Han et al. [8] demonstrated that graphene nanoplatelet-reinforced UHMWPE coatings significantly enhanced the corrosion resistance of 304L stainless steel. I.K. Aliyu et al. [9] determined that the optimal content of graphene nanoplatelets was approximately 1 wt.%; the UHMWPE coating containing 1 wt.% GNPs exhibited the greatest effectiveness in reducing the coefficient of friction and wear rate. In a 3.5% NaCl solution, the UHMWPE coating containing 2 wt.% GNPs exhibited the highest corrosion resistance. In turn, A. Golgoon et al. [10] demonstrated that nanoclay-modified polymer coatings exhibit considerably enhanced corrosion resistance.

The literature analysis indicates that carbon nanotubes and graphene have been extensively investigated in polymer composite coatings. However, studies on the mechanical properties and corrosion resistance of ceramic particle-reinforced UHMWPE-based composite coatings are still limited. Composite coatings based on UHMWPE are also regarded as promising materials for corrosion protection of metal surfaces. Nevertheless, despite the extensive investigation of the corrosion properties of UHMWPE-based composites produced by bulk pressing [11,12,13,14,15,16], the corrosion resistance of composite coatings obtained by flame spraying has not yet been fully studied. The investigation of the influence of various ceramic fillers on the corrosion properties of UHMWPE-based coatings is of considerable scientific and practical relevance.

In this context, the modification of UHMWPE-based composite coatings with hard ceramic particles represents an effective strategy for enhancing their wear resistance and mechanical strength. Therefore, the aim of this work was to develop flame-sprayed UHMWPE-based composite coatings reinforced with Al_2_O_3_ and SiC fillers with improved mechanical properties and abrasive wear resistance while preserving the structural integrity of the polymer matrix.

## 2. Materials and Methods

### 2.1. Materials

Commercial ultra-high molecular weight polyethylene (UHMWPE) powder produced by Nantong Yangba Polyethylene Co., Ltd. (Nantong Yangba Polyethylene Co., Ltd., Nantong, China) was used as the base material for coating preparation. The powder was characterized by a density of 930 kg/m^3^, a molecular weight of 2 × 10^6^ mol^−1^, a melting temperature of 135–150 °C, and a bulk density higher than 0.4 g/cm^3^ [17].

Aluminum oxide (Al_2_O_3_), with a molar mass of 101.96 g/mol and complying with TU 1791-003-36280340-2008, and black silicon carbide (SiC) powder, grade 51C, with an M28 (F400) grain size class and produced according to TU 2-036-972-85, were used as fillers. The filler contents were 5, 10, 15, and 20 wt.% [18,19].

To achieve a homogeneous composition of UHMWPE composites with Al_2_O_3_ and SiC fillers, the starting powders were mechanically mixed using a roller ball mill (XQM-4, Changsha Tencan Powder Technology Co., Ltd., Changsha, China). The powders were mixed for approximately 60 min at a mill rotation speed of 600 rpm. Ball milling was performed to ensure the homogeneous distribution of the filler particles within the polymer matrix.

### 2.2. Technology for Producing Coatings

**Figure 1:**
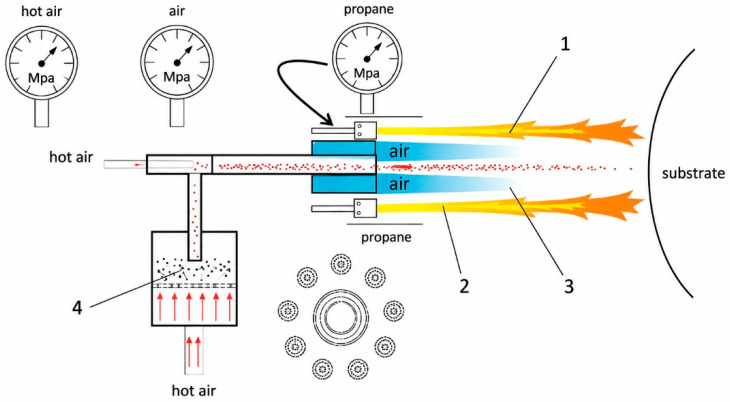
Schematic diagram of the flame spraying setup: 1—propane enters the nozzle, sprays, ignites under the influence of a heat source, and forms a torch; 2—an air damper that compresses the air flow with the powder; 3—supply of powder under pressure through the body of the torch; 4—unit for fluidization and powder preheating; adapted from [20].

UHMWPE-based composite coatings containing Al_2_O_3_ and SiC were produced on St3 steel substrates with dimensions of 25 mm × 25 mm × 2 mm by the flame spraying method shown in Figure 1. The spraying system was developed at the “Shygys Bastau” Technopark of S. Amanzholov East Kazakhstan University, Kazakhstan [20]. Prior to spraying, the steel substrate surfaces were sandblasted to provide the required roughness and enhance the adhesive strength of the coating. Propane served as the fuel gas, and air was used as the oxidizer. After the powder feeder was fully loaded, the pressure was gradually increased to form a fluidized bed with a height of 400 mm, as required by the technological process. Under these conditions, the height of the fluidized bed increased gradually as the pressure increased. The required fluidized bed height was achieved at a pressure of 0.13 MPa, with an air volumetric flow rate of 65 m^3^/h. The powder feeding rate was maintained at 1 kg/min, while the spraying distance was set to 800 mm. The selected technological parameters [21] provided uniform formation of the composite coatings and ensured their reliable bonding to the metal substrate surface.

### 2.3. Methods of Investigation

The microstructure, surface morphology, and porous structure characteristics of the initial Al_2_O_3_ and SiC powders and the UHMWPE-based coatings were investigated using an SEM 3200 scanning electron microscope (CIQTEK Co., Ltd., Hefei, China). The working distance from the sample surface to the lower part of the objective was 4.7 mm, while the accelerating voltage was set to 15 kV. Prior to SEM examination, the surfaces of the investigated samples were coated with a thin carbon (C) layer to ensure electrical conductivity.

The phase composition of the powders and the polymer composites was determined using an X’Pert PRO X-ray diffractometer (PANalytical B.V., Almelo, The Netherlands). During the analysis, the copper anode tube was operated at a voltage of 40 kV and a current of 30 mA. XRD measurements were carried out using Cu-Kα radiation (λ = 1.541 Å), and diffraction patterns were recorded within a specified 2θ angular range. The scanning step size was 0.02°, with a counting time of 0.5 s per step [22]. Phase analysis of the diffraction peaks in the obtained XRD patterns was performed using HighScore Plus software (Version 4.9, Malvern Panalytical B.V., Almelo, The Netherlands), Match! software (Version 3, Crystal Impact GbR, Bonn, Germany), and PowderCell software (Version 2.4, BAM Federal Institute for Materials Research and Testing, Berlin, Germany). Sample preparation, selection of acquisition parameters, and diffraction pattern analysis were carried out in accordance with the procedures described in the literature [23]. The calculations were performed using the 2θ diffraction angle range of 15–35°, which reflects the ratio of crystalline to amorphous phases in the polymer. The crystallinity degree was determined according to the following equation:

χ = Scr/Sa(1)

where

Scr—the area of the crystalline region, i.e., the area above the halo line;

Sa—the area of the amorphous region, i.e., the area below the halo line.

This method enables the assessment of the structural state of the composite materials and the influence of the filler on the crystalline structure of the polymer matrix.

Hardness measurements were performed using a FISCHERSCOPE HM2000 measurement system (Helmut Fischer GmbH, Sindelfingen, Germany) in accordance with DIN EN ISO 14577-1 [24]. According to the instrument specifications, the hardness measurement range was 0.001–120,000 N/mm^2^, the load-setting accuracy was 4 mg, the displacement resolution was 0.1 nm, and the measurement uncertainty was 2% of the measured value. The indenter approach velocity was 2 μm/s, and the applied load range was 0.1–2000 mN. The experimental data were processed using the instrument’s WIN-HCU software (Version 7.5, Helmut Fischer GmbH, Sindelfingen, Germany). A Vickers diamond indenter with a face angle of 136° was used for all measurements. A Vickers-type quadrangular diamond pyramid with an interfacial angle of 136° was used as the indenter. In this study, Martens hardness (HM) was chosen as the primary characteristic parameter. Because Martens hardness accounts for both plastic and elastic deformation of the material during measurement, it can be used to assess the mechanical properties of various materials. The measurements were carried out using the Vickers hardness method at a load of 300 mN and a dwell time of 20 s. This method allows the mechanical properties of the surface layer of coatings and composite materials to be evaluated with high accuracy.

The wear resistance of the polymer coatings was assessed based on the mass loss per unit time during abrasive wear testing. Abrasive wear tests were performed using an abrasive wear testing apparatus in accordance with the methodology described in [21]. The test conditions were as follows: a load of 18 N, exposure times of 10, 20, and 30 min, and corundum powder with a particle size below 100 μm as the abrasive material.

## 3. Results and Discussion

### 3.1. Results of SEM Analysis of the Powders

**Figure 2:**
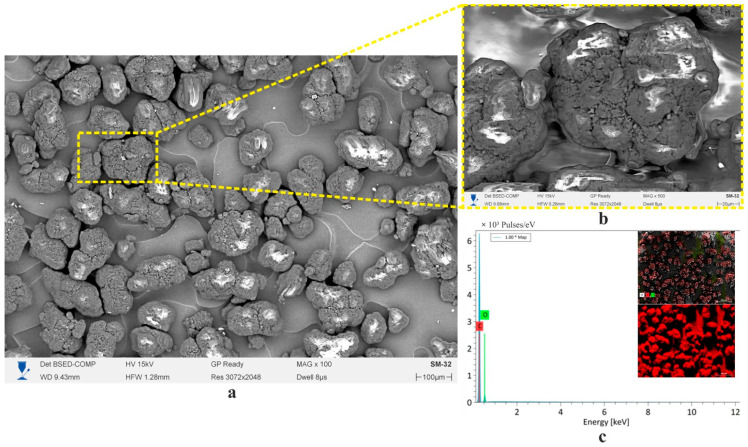
(**a**) SEM micrographs of the UHMWPE powder, (**b**) SEM image of the selected region, and (**c**) elemental analysis results of the UHMWPE powder.

**Figure 3:**
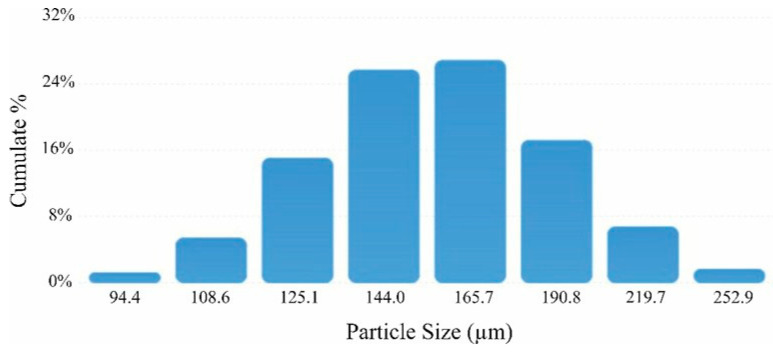
Particle size distribution analysis results of the UHMWPE powder.

Ultra-high molecular weight polyethylene (UHMWPE) in its initial state is a white powder material. The morphological features of the powder are presented in Figure 2. The SEM analysis revealed that the majority of the powder particles were approximately 150 μm in size. According to the granulometric analysis results presented in Figure 3, the particle sizes of the UHMWPE powder were distributed in the range of 90–250 μm. The predominant fraction of particles was distributed within the size range of 144–165 μm, which is in good agreement with the SEM results. This particle morphology enhances the technological properties of the powder and contributes to its uniform feeding during the spraying process. The elemental analysis carried out using energy-dispersive spectroscopy (EDS) revealed that the spectrum contained only carbon-related peaks, indicating that the UHMWPE powder did not contain any other chemical impurities.

**Figure 4:**
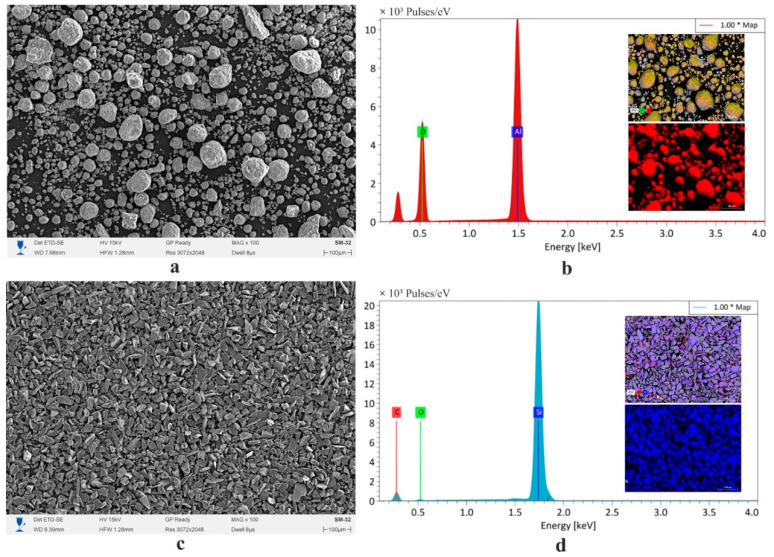
SEM micrograph of the initial Al_2_O_3_ powder (**a**), elemental analysis results of the Al_2_O_3_ powder (**b**), SEM micrograph of the initial SiC powder (**c**), and elemental analysis results of the SiC powder (**d**).

**Figure 5:**
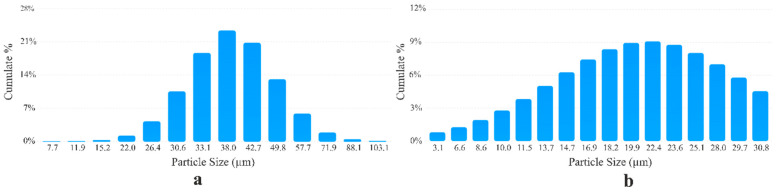
Particle size distribution analysis results of the initial Al_2_O_3_ powder (**a**) and SiC powder (**b**).

Figure 4 presents the SEM and EDS analysis results obtained to investigate the morphology and elemental composition of the Al_2_O_3_ and SiC fillers. According to the SEM analysis, the Al_2_O_3_ powder particles exhibited a polyhedral morphology. The mean particle size of the powder was approximately 30 μm. The SiC powder was mainly composed of particles with different geometric shapes. The mean particle size was approximately 14 μm. Figure 5 presents the particle size distribution of the fillers determined using a Winner 2005A laser particle size analyzer (Jinan Winner Particle Instrument Co., Ltd., Jinan, China). The average particle size (Dav) of the Al_2_O_3_ powder was 31.22 μm, whereas the granulometric analysis of the SiC powder showed an average particle size (Dav) of 16.46 μm. These results demonstrate good agreement between the SEM observations and the granulometric analysis data obtained using the two methods.

### 3.2. Results of SEM Analysis of the Coatings

**Figure 6:**
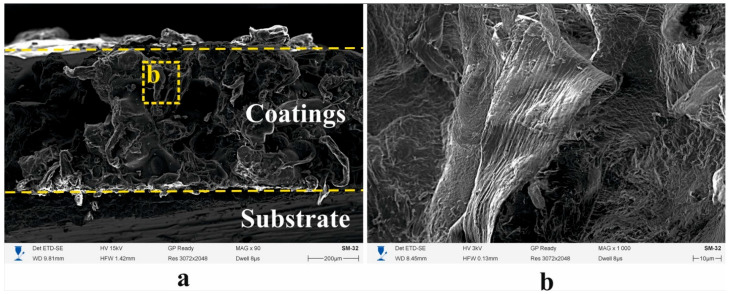
(**a**) Cross-sectional SEM micrograph of the UHMWPE coating and (**b**) magnified image of the selected region.

The morphology of the pure UHMWPE coating was examined by scanning electron microscopy (SEM) [25]. The low-magnification SEM image (90×) presented in Figure 6 indicates that the coating possesses a continuous structure. The coating exhibited a relatively uniform thickness distribution; however, the presence of individual voids may be related to the melting and coalescence of polymer particles during the coating formation process (Figure 6a).

**Figure 7:**
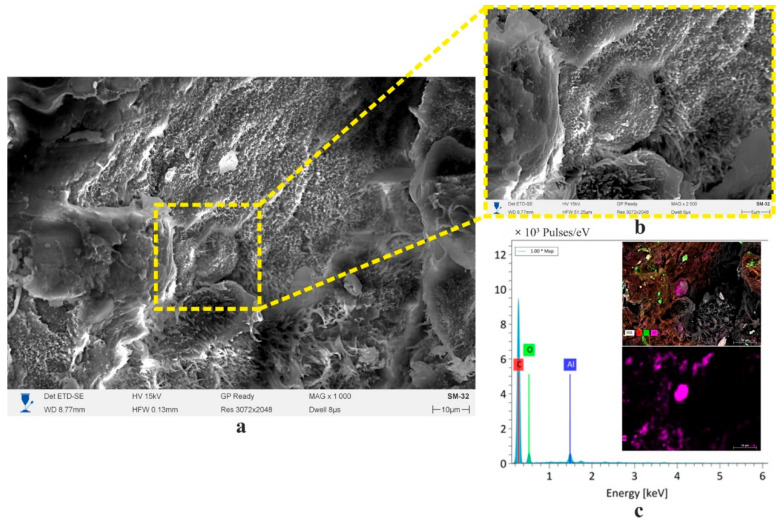
(**a**) SEM micrograph of the UHMWPE composite coating containing 5 wt.% Al_2_O_3_ filler, (**b**) magnified image of the selected region, and (**c**) EDS elemental analysis results.

**Figure 8:**
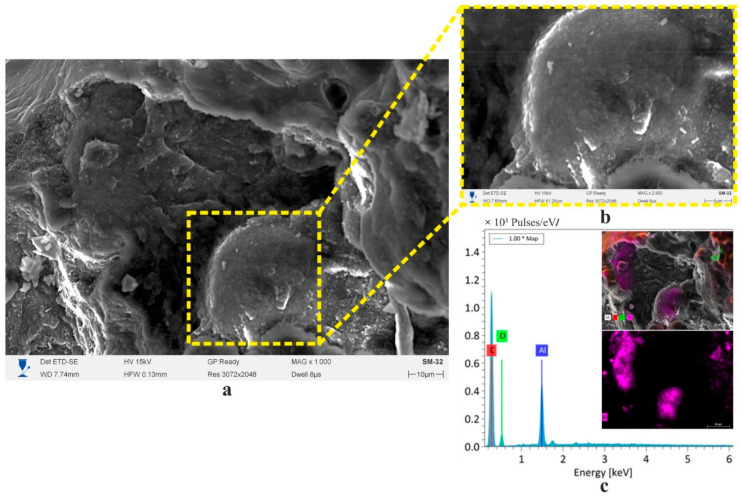
(**a**) SEM micrograph of the UHMWPE composite coating containing 10 wt.% Al_2_O_3_ filler, (**b**) magnified image of the selected region, and (**c**) EDS elemental analysis results.

**Figure 9:**
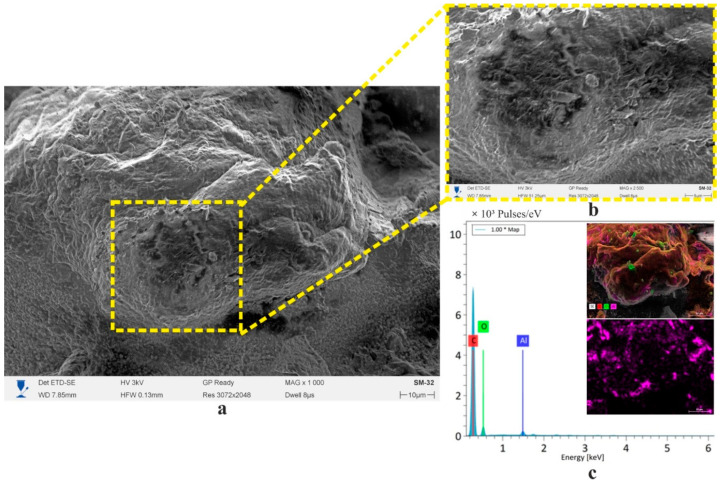
(**a**) SEM micrograph of the UHMWPE composite coating containing 15 wt.% Al_2_O_3_ filler, (**b**) magnified image of the selected region, and (**c**) EDS elemental analysis results.

**Figure 10:**
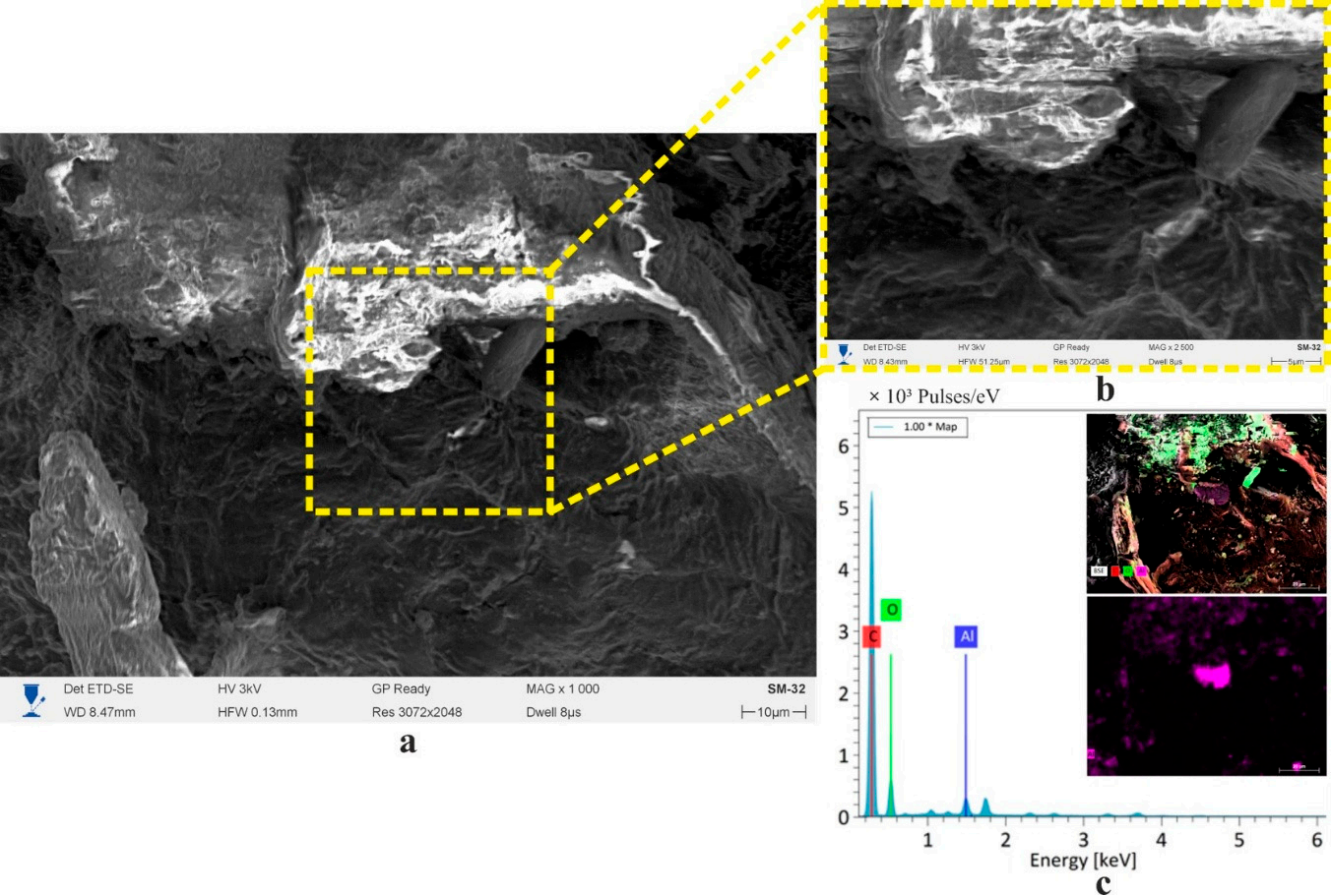
(**a**) SEM micrograph of the UHMWPE composite coating containing 20 wt.% Al_2_O_3_ filler, (**b**) magnified image of the selected region, and (**c**) EDS elemental analysis results.

SEM images and EDS elemental mapping were used to assess the distribution behavior of the fillers within the polymer matrix and their interaction characteristics with the UHMWPE matrix. As shown in Figure 7, the SEM images of the UHMWPE coating containing 5 wt.% Al_2_O_3_ demonstrated a uniform distribution of filler particles within the polymer matrix. Aluminum-containing particles were observed in the coating structure, while the Al_2_O_3_ particles were well dispersed within the matrix volume. No clearly defined interfacial separation regions were observed between the filler particles and the polymer matrix. The EDS spectrum confirmed the presence of carbon, oxygen, and aluminum in the coating. The detection of aluminum and oxygen confirms that the Al_2_O_3_ filler was successfully incorporated into the coating structure. As shown in Figure 8, although most of the filler particles in the coating containing 10 wt.% Al_2_O_3_ were uniformly distributed within the polymer matrix, localized regions of particle agglomeration were observed. EDS elemental mapping revealed localized accumulation of Al, suggesting partial agglomeration of Al_2_O_3_ particles within the polymer matrix. Nevertheless, due to the low degree of agglomeration, the overall structural homogeneity of the coating was preserved. As shown in Figure 9, the SEM images of the coating containing 15 wt.% Al_2_O_3_ demonstrated the distribution of filler particles within the polymer matrix. The majority of the particles were incorporated into the matrix, with no clearly defined interfacial separation regions observed around them. EDS elemental mapping confirmed the distribution of aluminum and oxygen across the examined region [26]. This suggests that the Al_2_O_3_ particles were successfully incorporated into the coating structure and established satisfactory interfacial bonding with the polymer matrix. No distinct large agglomerates were identified within the examined region. As shown in Figure 10, the cross-sectional SEM images of the composite coating containing 20 wt.% Al_2_O_3_ reveal a significant increase in the number of filler particles. Large Al_2_O_3_ particles were clearly observed in the examined region, indicating their close interfacial contact with the polymer matrix. Compared with the coatings containing 5–15 wt.% Al_2_O_3_, this sample exhibits a higher fraction of the filler phase, making the presence of ceramic particles in the coating structure more pronounced. Elemental mapping revealed the distribution of Al and O within the examined region, confirming the successful incorporation of aluminum oxide into the composite structure.

**Figure 11:**
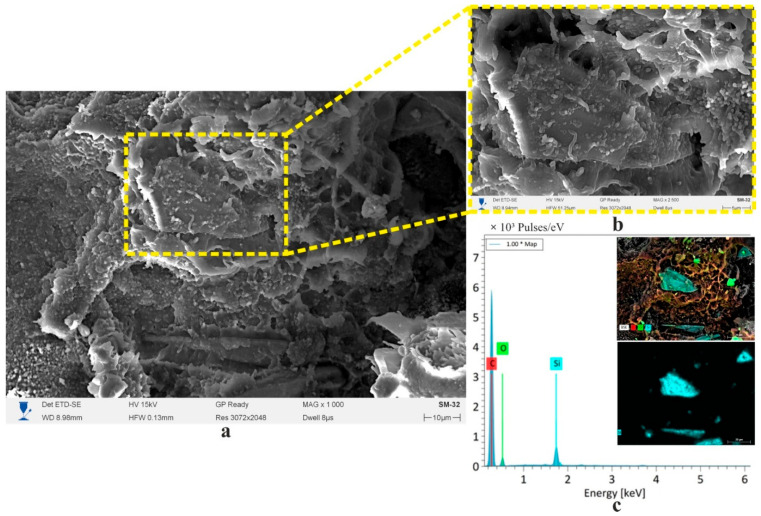
SEM micrograph of the UHMWPE composite coating containing 5 wt.% SiC filler (**a**), magnified SEM image of the selected region (**b**), and EDS elemental analysis results (**c**).

**Figure 12:**
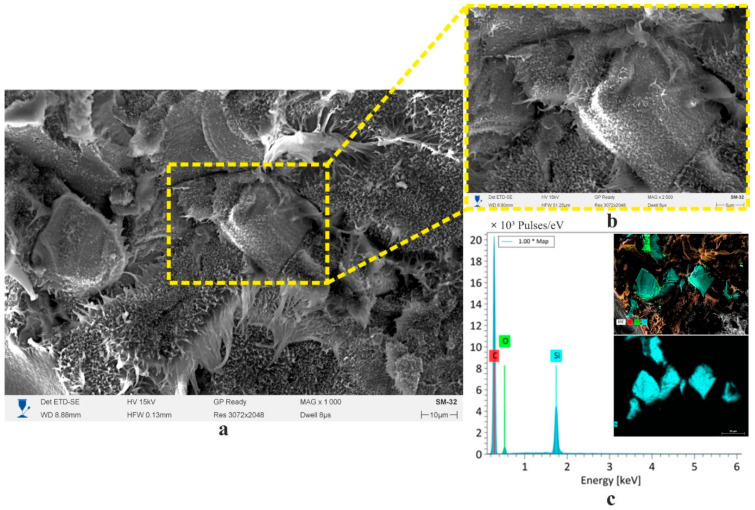
SEM micrograph of the UHMWPE composite coating containing 10 wt.% SiC filler (**a**), magnified SEM image of the selected region (**b**), and EDS elemental analysis results (**c**).

**Figure 13:**
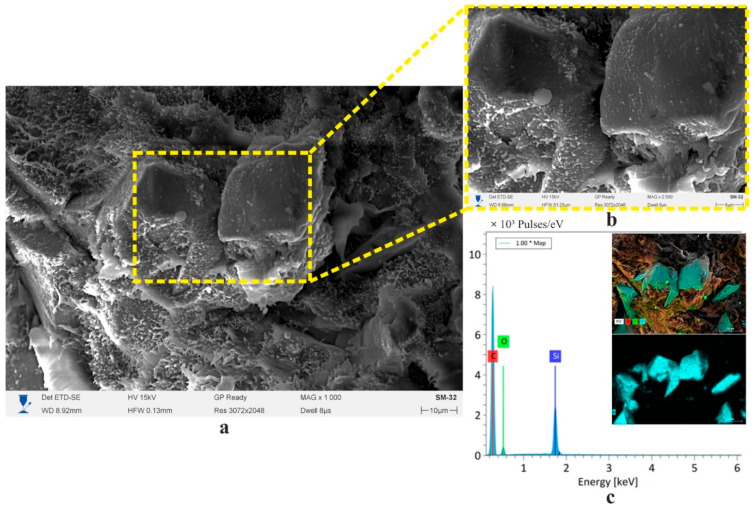
SEM micrograph of the UHMWPE composite coating containing 15 wt.% SiC filler (**a**), magnified SEM image of the selected region (**b**), and EDS elemental analysis results (**c**).

**Figure 14:**
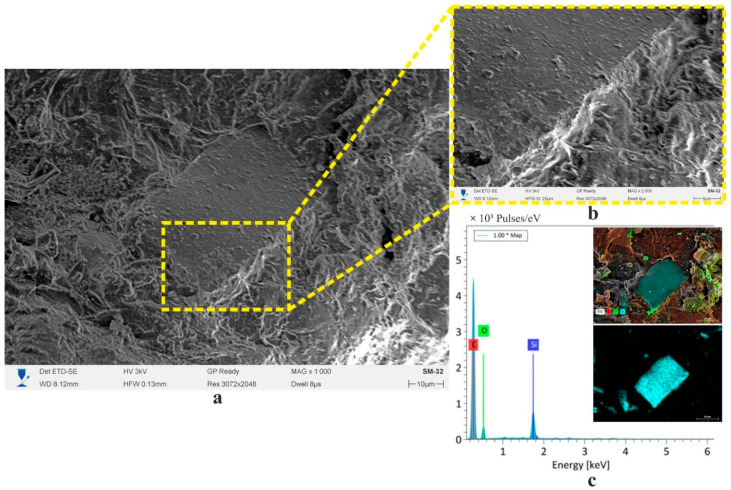
SEM micrograph of the UHMWPE composite coating containing 20 wt.% SiC filler (**a**), magnified SEM image of the selected region (**b**), and EDS elemental analysis results (**c**).

As shown in Figure 11, the cross-sectional SEM images of the UHMWPE composite coating containing 5 wt.% SiC reveal SiC particles embedded within the polymer matrix. The SiC particles were predominantly dispersed as individual particles and exhibited good bonding with the polymer matrix. The magnified region shows that the particles are surrounded by the polymer phase, suggesting their effective incorporation into the coating structure. Furthermore, the small size of the SiC particles facilitated their uniform distribution throughout the matrix volume. As shown in Figure 12, the SEM images of the composite coating containing 10 wt.% SiC demonstrated an increase in the fraction of filler particles within the coating structure. SiC particles were distributed across various regions of the polymer matrix, and the matrix retained its continuous structure around the particles. The magnified image reveals close interfacial contact between the SiC particle surfaces and the polymer, indicating strong anchoring of the filler within the coating structure. As shown in Figure 13, the images of the composite coating containing 15 wt.% SiC clearly demonstrate the presence of ceramic filler particles. In the magnified region, the SiC particles exhibited well-defined boundaries and remained as separate structural elements within the polymer matrix. With increasing filler content, the fraction of the ceramic phase in the coating structure was observed to increase. Furthermore, the polymer matrix retained its continuous structure around the SiC particles, demonstrating their effective incorporation into the coating structure. As shown in Figure 14, SiC particles are clearly visible in the cross-sectional SEM images of the composite coating containing 20 wt.% SiC. The magnified image indicates that the particle preserved its well-defined geometric shape and was positioned at the interface with the polymer matrix. The EDS spectrum confirmed the presence of oxygen and silicon. The particle preserved the main characteristics of its initial morphology and was embedded within the matrix volume.

SEM analysis revealed that the flame-sprayed UHMWPE coatings reinforced with Al_2_O_3_ and SiC exhibited a homogeneous microstructure. During the spraying process, the molten UHMWPE encapsulated the ceramic particles, resulting in their incorporation into the polymer matrix. EDS analysis confirmed the presence and distribution of both fillers throughout the coating. Owing to their smaller particle size, SiC particles were dispersed more uniformly within the matrix, whereas Al_2_O_3_ particles showed a tendency toward localized clustering at higher filler contents. Nevertheless, no cracks, coating delamination, or pronounced agglomeration were observed in any of the investigated samples.

### 3.3. Results of X-Ray Phase Analysis of the Coatings

X-ray phase analysis was carried out for UHMWPE-based coatings containing Al_2_O_3_ and SiC fillers. Similar diffraction patterns of UHMWPE were also reported in [27]. According to the literature [28], UHMWPE is characterized by an orthorhombic crystal lattice. These results demonstrate that the incorporation of Al_2_O_3_ and SiC fillers does not disturb the initial crystalline structure of the polymer matrix, while the flame spraying process preserves the structural stability of UHMWPE. In all compositions containing Al_2_O_3_ and SiC fillers, no significant shifts in the positions of the main diffraction maxima were observed.

**Figure 15:**
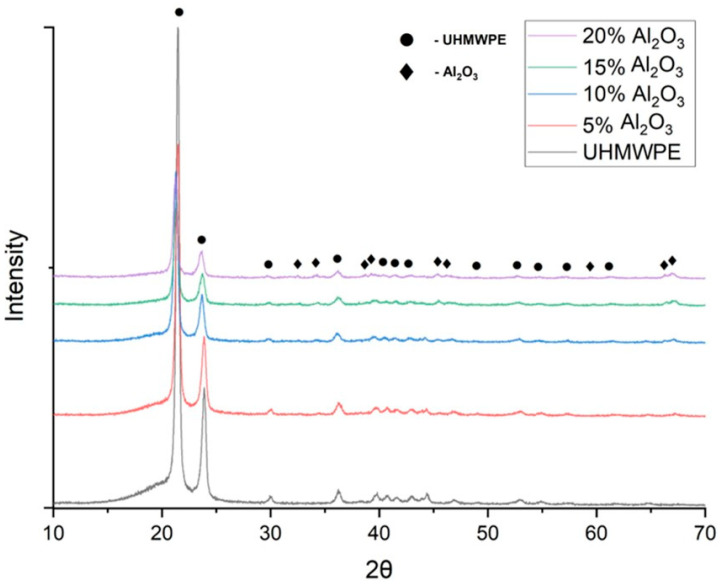
X-ray diffraction patterns of pure UHMWPE and composite coatings containing 5–20 wt.% Al_2_O_3_.

As shown in Figure 15, with increasing Al_2_O_3_ filler content, the intensity of the main diffraction peaks characteristic of UHMWPE gradually decreases in the composites. This behavior is attributed to the increased fraction of the Al-containing phase in the composite and the corresponding decrease in the relative amount of the polymer phase. Furthermore, the intensity of the diffraction reflections characteristic of the α-Al_2_O_3_ (corundum) phase, located in the 30–70° range, increases, confirming the preservation of filler particles in the composite structure and the increase in their content.

**Figure 16:**
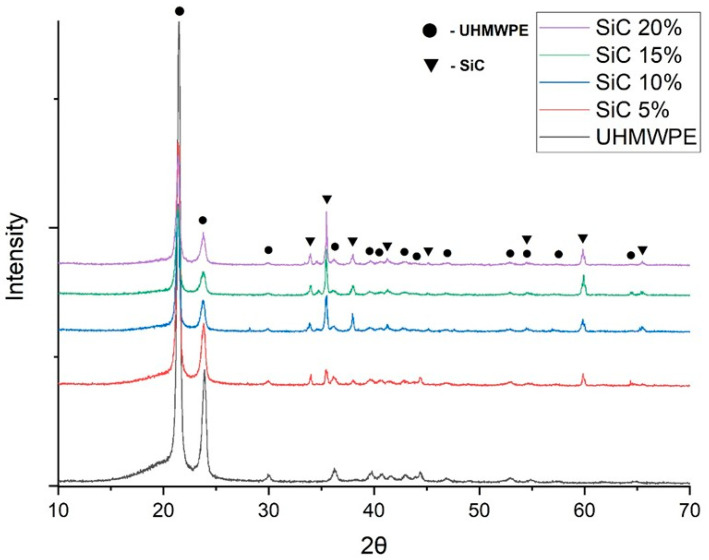
X-ray diffraction patterns of pure UHMWPE and composite coatings containing 5–20 wt.% SiC.

Figure 16 shows that, with increasing SiC filler content, the relative intensity of the main diffraction peaks characteristic of UHMWPE decreases, whereas the reflections corresponding to the SiC phase become more pronounced. This demonstrates that SiC particles were successfully incorporated into the composite structure and distributed throughout the matrix volume. Furthermore, the high hardness and thermal stability of SiC crystals promote the distinct appearance of their X-ray diffraction reflections.

The X-ray diffraction patterns were analyzed, and the degree of crystallinity was calculated using HighScore Plus software (Version 4.9, Malvern Panalytical B.V., Almelo, The Netherlands). The initial UHMWPE sample exhibited a crystallinity degree of 77%. The incorporation of Al_2_O_3_ and SiC fillers was found to affect the crystallinity of the polymer matrix to varying degrees. As shown in Table 1, the crystallinity degree decreased to 64% for the Al_2_O_3_-containing sample, while it remained at 71% for the SiC-containing sample. Therefore, the SiC filler more effectively maintains the crystalline structure of the UHMWPE matrix, while Al_2_O_3_ particles exert a stronger influence on the crystallization process of the polymer chains.

### 3.4. Microhardness Test Results of the Composite Coatings

**Figure 17:**
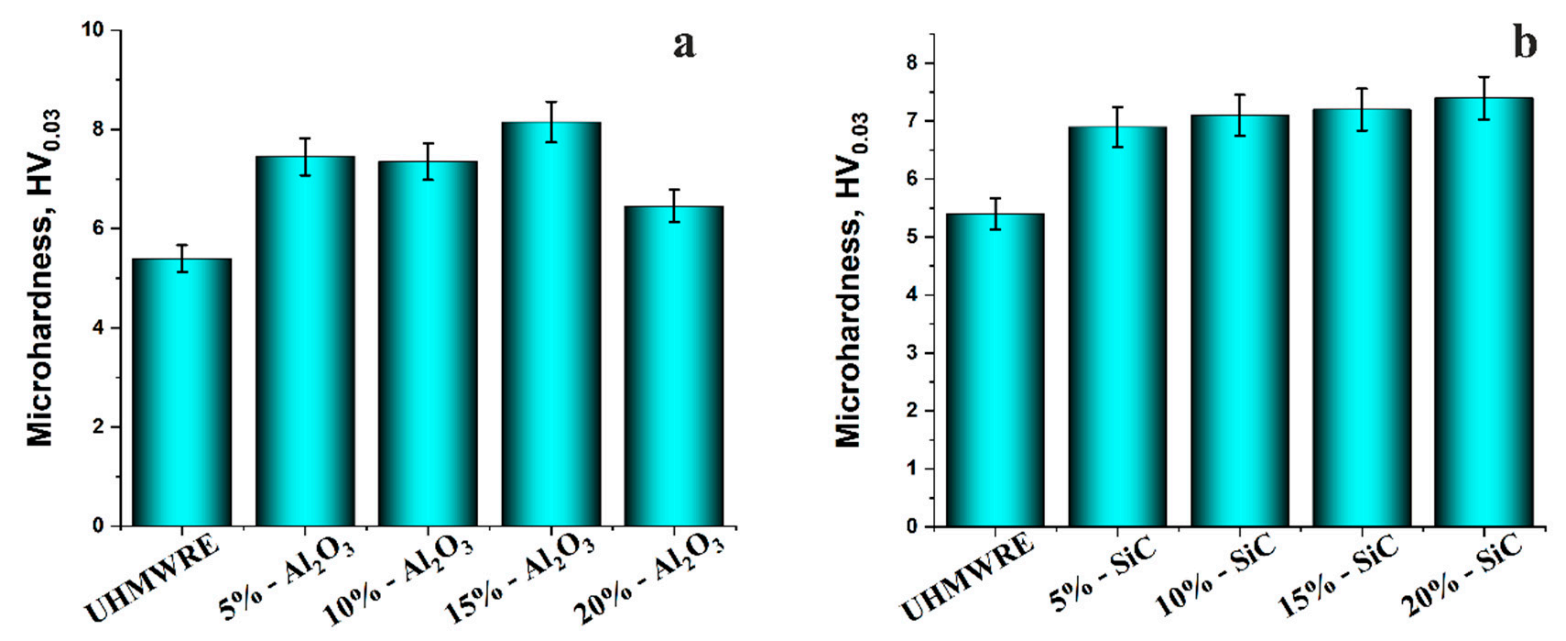
Vickers microhardness of UHMWPE-based composite coatings reinforced with Al_2_O_3_ (**a**) and SiC (**b**).

The comparison of the influence of Al_2_O_3_ and SiC fillers on the microhardness of UHMWPE-based composite coatings demonstrated that both fillers contributed to an increase in the hardness of the polymer matrix. The initial UHMWPE coating exhibited a microhardness of 5.4 ± 0.3 HV_0.03_, whereas this value increased for all samples containing fillers. As shown in Figure 17a, the incorporation of Al_2_O_3_ led to a maximum microhardness of 8.2 ± 0.5 HV_0.03_ at 15 wt.%, while a further increase in filler content to 20 wt.% resulted in a decrease to 6.5 ± 0.9 HV_0.03_. This decrease may be attributed to the agglomeration of filler particles at higher concentrations and their non-uniform distribution within the polymer matrix. Figure 17b shows that, in the case of the SiC filler, the microhardness gradually increased with increasing filler content, reaching a maximum value of 7.4 ± 0.4 HV_0.03_ at 20 wt.%.

X-ray diffraction analysis revealed that the incorporation of Al_2_O_3_ and SiC fillers resulted in a slight decrease in the degree of crystallinity of UHMWPE. Nevertheless, the microhardness of the composite coatings increased. This improvement cannot be attributed to changes in crystallinity but rather to the reinforcing effect of the ceramic fillers and the strong interfacial bonding formed between the fillers and the polymer matrix. SEM observations confirmed that the filler particles were uniformly distributed throughout the matrix and exhibited good interfacial adhesion with the polymer. Consequently, the presence of hard ceramic particles within the UHMWPE matrix contributed significantly to the enhancement of the microhardness of the composite coatings.

### 3.5. Abrasive Wear Test Results of the Composite Coatings

**Figure 18:**
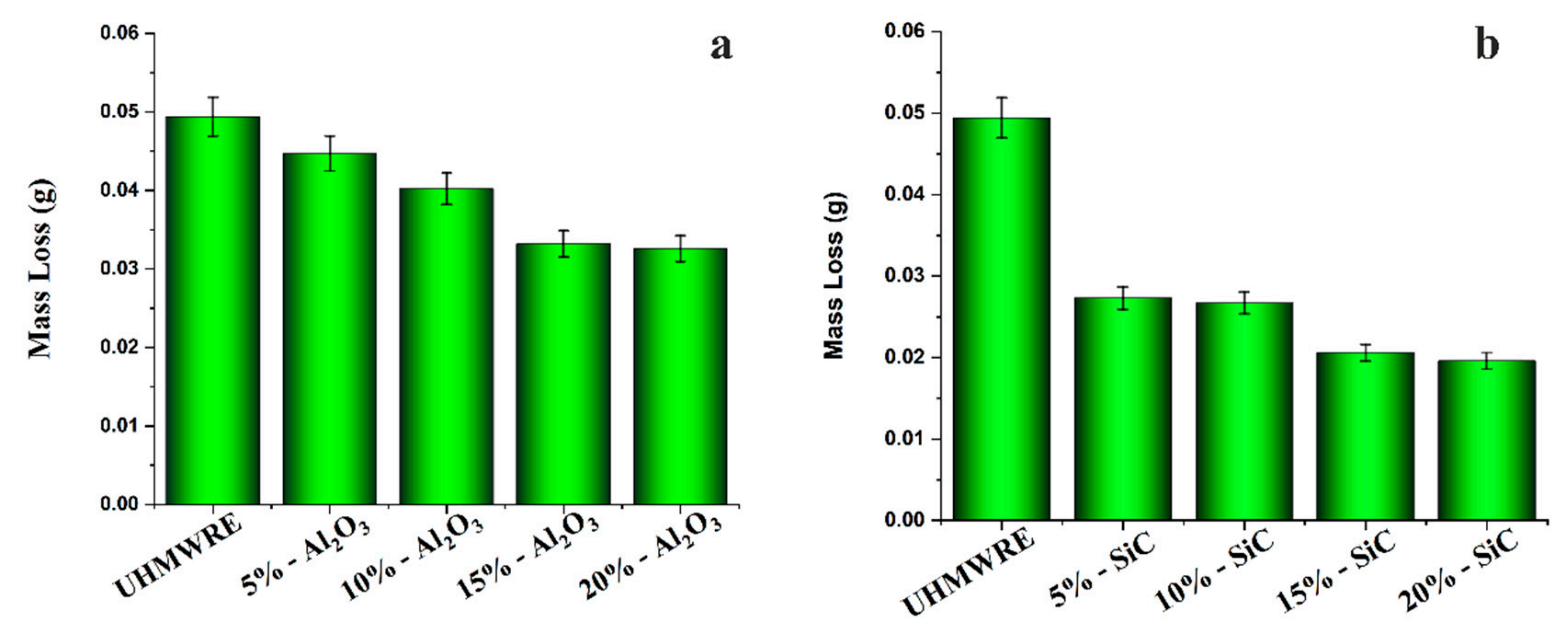
Abrasive wear test results of neat UHMWPE and UHMWPE-based composite coatings containing 5–20 wt.% Al_2_O_3_ (**a**) and SiC (**b**).

The evaluation of the effects of Al_2_O_3_ and SiC fillers on the abrasive wear resistance of UHMWPE-based composite coatings demonstrated that both fillers reduced mass loss and enhanced the wear resistance of the coatings. Table 2 summarizes the abrasive wear test results of UHMWPE and UHMWPE composite coatings containing 5–20 wt.% Al_2_O_3_ and SiC fillers. The initial UHMWPE sample exhibited a mass loss of approximately 0.05 g. Figure 18a shows that the incorporation of Al_2_O_3_ filler reduced the mass loss to 0.045–0.033 g. The minimum mass loss was observed for the sample containing 20 wt.% Al_2_O_3_, indicating a substantial improvement in wear resistance compared with the initial UHMWPE coating. This behavior can be attributed to the reinforcement of the polymer matrix by hard ceramic particles and the enhanced resistance of the coating to the action of abrasive particles. Figure 18b shows that, in the SiC-containing samples, the mass loss decreased further, reaching values in the range of 0.03–0.02 g. The minimum mass loss was recorded for the sample containing 20 wt.% SiC. This suggests that SiC particles more effectively mitigate abrasive action owing to their high hardness and excellent wear resistance [29]. The abrasive wear and microhardness results showed a positive correlation with the degree of crystallinity. Coatings with higher microhardness exhibited lower mass loss during abrasive wear tests, indicating that the incorporation of hard ceramic particles effectively enhanced the wear resistance of the UHMWPE-based composite coatings.

## 4. Conclusions

This study investigates the microstructure and performance of flame-sprayed UHMWPE-based composite coatings reinforced with 5–20 wt.% Al_2_O_3_ and SiC ceramic fillers. SEM and EDS analyses confirmed the successful incorporation of both ceramic fillers into the UHMWPE matrix. The filler particles were uniformly distributed throughout the coating, encapsulated by the polymer matrix, and exhibited strong interfacial bonding with the surrounding polymer, demonstrating the suitability of the flame spraying process for producing homogeneous composite coatings. X-ray diffraction analysis revealed that the incorporation of Al_2_O_3_ and SiC did not significantly alter the crystalline structure of UHMWPE. The degree of crystallinity ranged from 64% to 77% for the Al_2_O_3_-filled coatings and from 71% to 76% for the SiC-filled coatings. The addition of ceramic fillers significantly improved the mechanical and tribological properties of the coatings. The highest microhardness was achieved for the coating containing 15 wt.% Al_2_O_3_, reaching 8.2 ± 0.5 HV_0.03_, which represents a 50.9% increase compared with the neat UHMWPE coating. For the SiC-filled coatings, the maximum microhardness was 7.4 ± 0.4 HV_0.03_ at 20 wt.% SiC, corresponding to a 37.0% improvement over the unfilled coating. Abrasive wear tests further demonstrated that both fillers effectively enhanced the wear resistance of the coatings. The minimum mass loss for the Al_2_O_3_-filled coatings was 0.0326 g, corresponding to an approximately 1.5-fold improvement in wear resistance compared with neat UHMWPE. In contrast, the coating containing 20 wt.% SiC exhibited the lowest mass loss of 0.0196 g, representing an approximately 2.5-fold increase in wear resistance. Based on these results, 15 wt.% Al_2_O_3_ is considered the optimal composition for maximizing the mechanical performance of UHMWPE-based composite coatings, whereas 20 wt.% SiC provides the highest abrasive wear resistance owing to its combination of the lowest mass loss and superior preservation of the UHMWPE crystalline structure.

## Figures and Tables

**Table 1:** Crystallinity degree of UHMWPE in the composite coatings.

Fillers	Initial UHMWPE	Filler Content, wt.%			
		5 wt.%	10 wt.%	15 wt.%	20 wt.%
Al_2_O_3_-containing sample	77	75	73	67	64
SiC-containing sample	77	76	72	75	71

**Table 2:** Abrasive wear test results of UHMWPE and UHMWPE composite coatings containing 5–20 wt.% Al_2_O_3_ and SiC fillers.

№	Sample		Testing Time, min	Initial Weight, g	Final Weight, g	Abrasive Wear (Mass Loss), g
1.	UHMWPE		30	6.2931	6.2437	0.0494
2.	Al_2_O_3_	5%	30	6.3525	6.3078	0.0447
		10%		6.3928	6.3526	0.0402
		15%		6.4693	6.4361	0.0332
		20%		6.4891	6.4565	0.0326
3.	SiC	5%	30	6.3312	6.3039	0.0273
		10%		6.3831	6.3564	0.0267
		15%		6.4347	6.4141	0.0206
		20%		6.4909	6.4713	0.0196

## Data Availability

The original contributions presented in the study are included in this article; further inquiries can be directed to the corresponding author.
